# Distinct mechanisms of *Drosophila* CRYPTOCHROME-mediated light-evoked membrane depolarization and in vivo clock resetting

**DOI:** 10.1073/pnas.1905023116

**Published:** 2019-10-28

**Authors:** Lisa S. Baik, David D. Au, Ceazar Nave, Alexander J. Foden, Wendy K. Enrriquez-Villalva, Todd C. Holmes

**Affiliations:** ^a^Department of Physiology and Biophysics, School of Medicine, University of California, Irvine, CA 92697

**Keywords:** cryptochrome, flavoprotein, phototransduction, *Drosophila*, circadian clock

## Abstract

*Drosophila* CRYPTOCHROME (dCRY) mediates electrophysiological depolarization and circadian clock resetting in response to blue or ultraviolet (UV) light. These light-evoked biological responses operate at different timescales and possibly through different mechanisms. Whether electron transfer down a conserved chain of tryptophan residues underlies biological responses following dCRY light activation has been controversial. To examine these issues in in vivo and in ex vivo whole-brain preparations, we generated transgenic flies expressing tryptophan mutant dCRYs in the conserved electron transfer chain and then measured neuronal electrophysiological phototransduction and behavioral responses to light. Electrophysiological-evoked potential analysis shows that dCRY mediates UV and blue-light–evoked depolarizations that are long lasting, persisting for nearly a minute. Surprisingly, dCRY appears to mediate red-light–evoked depolarization in wild-type flies, absent in both *cry-null* flies, and following acute treatment with the flavin-specific inhibitor diphenyleneiodonium in wild-type flies. This suggests a previously unsuspected functional signaling role for a neutral semiquinone flavin state (FADH^•^) for dCRY. The W420 tryptophan residue located closest to the FAD-dCRY interaction site is critical for blue- and UV-light–evoked electrophysiological responses, while other tryptophan residues within electron transfer distance to W420 do not appear to be required for light-evoked electrophysiological responses. Mutation of the dCRY tryptophan residue W342, more distant from the FAD interaction site, mimics the *cry-null* behavioral light response to constant light exposure. These data indicate that light-evoked dCRY electrical depolarization and clock resetting are mediated by distinct mechanisms.

## Significance

Phototransduction is relatively well characterized in the eyes and other external photoreceptors in animals for image-forming vision. Much less understood are phototransduction mechanisms in noneye photosensitive cells that include central brain neurons that mediate the detection of nonimage-forming light irradiance detection, which allows animals to reset circadian clock timing and modulate light-evoked arousal, phototaxis, and avoidance. Here we provide a detailed biophysical characterization of *Drosophila* CRYPTOCHROME (dCRY)-mediated phototransduction, including dCRY expressed in central brain neurons for light-evoked membrane electrical changes in depolarization and action potential firing frequency and behavioral responses to constant light.

CRYPTOCHROME (CRY) is a highly conserved and evolutionarily ancient flavoprotein expressed widely in prokaryotes and eukaryotes. Light-sensitive *Drosophila* CRYPTOCHROMEs (dCRYs) exhibit 2 absorbance peaks at 365 nm [ultraviolet (UV) light] and 450 nm (blue light) both in vitro and in cells (1–3). Light activation of dCRY initiates a relatively slow (∼1 h) irreversible process of ubiquitin-mediated degradation of the clock protein TIMELESS (TIM) and dCRY itself, thus resetting the circadian clock (4–8). More recently, dCRY was discovered to mediate rapid membrane depolarization (τ_on_ ∼ 100 ms) and an increased spontaneous action potential firing rate in the lateral ventral neurons (LNvs) in response to blue and UV light (9–12). The mechanisms for light-activated dCRY leading to TIM degradation/clock resetting and membrane depolarization may differ based on timing of the response onset and reversibility/irreversibility following light exposure. For clock resetting, the light activation of dCRY leads to displacement of its short helical C-terminal tail (CTT) allowing TIM binding, which triggers the JETLAG (JET) ubiquitin-ligase–mediated targeting of TIM for proteolytic degradation (6, 8, 13–16). In contrast, dCRY-mediated light-evoked depolarization and increased action potential firing frequency (FF) are robust in the genetic absence of TIM and in dCRY mutants lacking the C terminus (10).

**Fig. 1.**
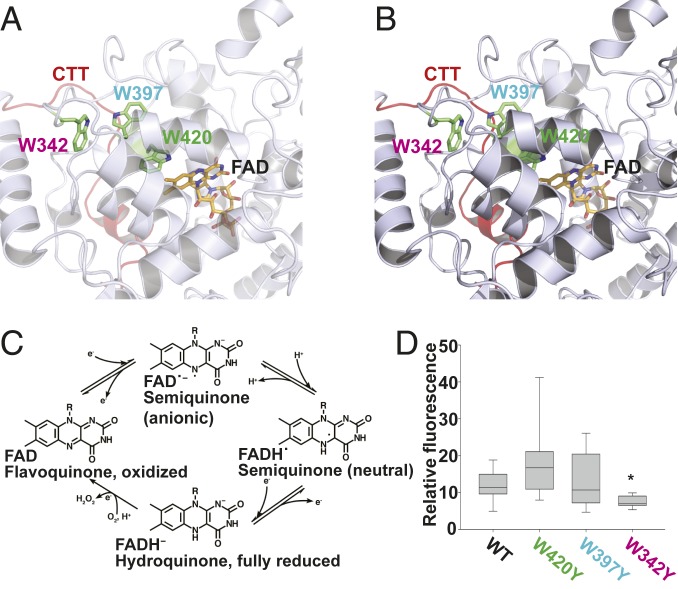
Site-directed mutagenesis of *cryptochrome* expressed in vivo. (*A* and *B*) Structural models of CRYPTOCHROME bound to the FAD cofactor (Protein Data Bank accession 4GU5). (*C*) Different states of the FAD redox cycle. (*D*) Quantification of the relative fluorescence level of no mutation control (WT dCRY, *n* = 11) or transgenic point-mutant dCRY (W420Y, *n* = 10; W397Y, *n* = 10; W342Y, *n* = 11) expression in the adult brain. Box plot represents median (line within box), upper and lower quartiles (box), and minimum and maximum (whiskers) values. *P < 0.05 vs. WT dCRY.

dCRY photoactivation requires the flavin adenine dinucleotide (FAD) cofactor. The *crybaby* mutation in the FAD-binding site of dCRY impairs FAD binding to dCRY (5). Both light-evoked circadian clock resetting and electrophysiological responses are severely attenuated in *crybaby* mutant flies (5, 9). Upon light exposure, the dCRY FAD cofactor is photoreduced from an oxidized state to an anionic semiquinone (FAD^•-^) state (Fig. 1*C*) (3, 17, 18). In biophysical assays of CRYs, including those from plants and related photolyase (PL) DNA repair enzymes, the light-excited FAD cofactor is photoreduced by electron transfer along a chain of highly conserved tryptophan (Trp; W) residues linking the FAD cofactor with the protein surface (1, 2, 19, 20). Three conserved Trp residues were initially characterized as the “Trp-triad molecular wire” responsible for reductively quenching light-excited FAD via sequential electron transfer (Fig. 1 *A* and *B*, based on structure from refs. 20 and 21). More recently, a fourth Trp residue was identified as part of the Trp molecular chain in dCRY, a “Trp-tetrad” (19, 20, 22–24). These Trp residues create an electron transfer chain through which FAD is reduced, balanced by the deprotonation of solvent-exposed Trps down the chain (25). For the circadian clock resetting mechanism, there is consensus that the light activation of dCRY-FAD eventually leads to conformational changes in the dCRY CTT that promote dCRY interaction with TIM, thus triggering proteolytic degradation. The functional significance of electron transfer along the Trp-tetrad chain and the relative ground versus excited redox state of FAD-triggering biological responses is controversial (24, 26–28). Light-evoked dCRY-mediated membrane depolarization in *Drosophila* lateral ventral (LNv) neurons depends on potassium channel heteromultimeric complexes consisting of redox-sensing cytoplasmic potassium beta (Kvβ) HYPERKINETIC (HK) subunits and ion-conducting voltage-gated potassium alpha (Kvα) ether-a-go-go family subunits (11, 12). However, the molecular mechanism of redox coupling between dCRY and FAD leading to electrophysiological membrane potential changes remains unclear. To explore the functional importance of Trp-mediated electron transfer for functional electrophysiological and behavioral light responses, we created transgenic flies that express Trp to tyrosine (Tyr; Y) mutations in the dCRY Trp-triad.

## Results

### Generation of Tryptophan Mutant dCRY Transgenic Flies.

We generated transgenic UAS- fly lines to express single-point Trp-to-Tyr mutant dCRYs and then used the *cry24-Gal4* driver line (29) to express wild-type (WT) and mutant dCRYs in dCRY-expressing neurons in a *cry-null* mutant fly genetic background (30). The targeted expression of WT and mutant dCRY was monitored by an N-terminal fusion protein of dCRY and enhanced-GFP, which also informs protein expression levels. Because tryptophan has a large, bulky ring structure we individually replaced Trp residues with Tyr, a large aromatic residue, rather than a small residue such as an alanine to prevent collapse of the dCRY structure. Trp-to-Tyr substitution is expected to perturb but not abolish electron transfer because, in contrast to phenylalanine (Phe, F), Tyr is still redox active but differs from Trp in its protonation properties. We targeted 3 different Trp residues near the FAD-binding site of dCRY that play a role in electron transfer: W342, W397, and W420, shown in the dCRY high-resolution structure and ranked from farthest to closest to the FAD-binding site (Fig. 1 *A* and *B* and *SI Appendix*, Fig. S1 *A* and *B*). Four possible flavin redox states are depicted in Fig. 1*C*. The oxidized FAD and the anionic semiquinone states (FAD^•-^) have been detected biophysically in purified dCRY protein (3, 17, 18). We also consider the unexpected possibility that neutral semiquinone state flavin (FADH^•^) may also contribute to electrophysiological light responses based on several lines of experimental evidence (see below). Expression of all dCRY transgenics and no-UAS negative control was confirmed by confocal imaging of freshly dissected transgenic adult fly brains and quantified for equivalency of expression levels (Fig. 1*D* and *SI Appendix*, Fig. S2). W342Y dCRY protein expression is readily measured but, compared to WT dCRY, is semiquantitatively lower (Fig. 1*D* and *SI Appendix*, Fig. S2). All other mutant dCRYs express at levels comparable to that of WT dCRY (Fig. 1*D* and *SI Appendix*, Fig. S2).

### Transgenic WT dCRY Rescues Light-Evoked Depolarization in a FAD-Specific Manner.

We used a highly sensitive and time-resolved light-evoked potential method to capture membrane potential changes using whole-cell patch-clamp recordings of large-lateral ventral neurons (l-LNv) in an ex vivo preparation of adult fly whole brains. The light-evoked potential protocol consists of a baseline prestimulus recording of membrane potential followed by 5 s of narrow emission spectra light-emitting diode (LED) light stimuli (150 μW/cm^2^, controlled by TTL trigger) under current-clamp mode. Based on our previous studies of UV and blue-light–evoked increases in l-LNv action potential FF, 150 μW/cm^2^ (1.5 W/m^2^) light intensity is relatively low but above the reliable threshold for evoking increased FF (10, 12). Recordings were time-locked to light onset and offset in a repetitive manner (minimum 5 light stimuli per cell per color, 5 to 29 cells) and then averaged. Based on empirical recording data, we employed a 95-s interval between light stimuli to achieve full recovery of the membrane potential baseline state. The evoked potential protocol averages out individual action potentials and random electrical fluctuations to provide a robust membrane voltage light response (10).

Electrophysiological responses to low-intensity–matched UV (365 nm LED, 150 μW/cm^2^), blue (450 nm LED, 150 μW/cm^2^), and red light (630 nm LED, 150 μW/cm^2^) were measured in l-LNv recordings from transgenic flies expressing WT dCRY and from *cry-null* negative control flies. The dCRY red light responses were initially measured as negative controls, an assumption based on earlier work, including with purified protein, indicating that the flavin cofactor in dCRY does not reach a FADH^•^ neutral semiquinone state capable of red light absorbance and signaling (3, 17–19, 26).

**Fig. 2.**
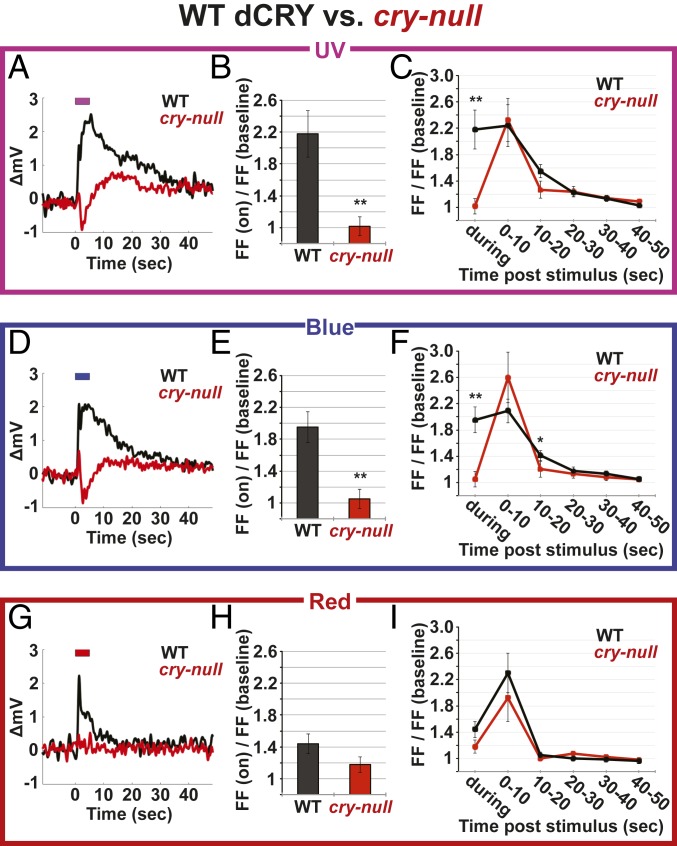
WT dCRY control rescues electrophysiological response to light, while *cry-null* flies have reduced responses during lights-on. l-LNv electrophysiological light responses of no mutation (WT dCRY) control (black) (WT dCRY: UV *n* = 19; blue *n* = 29; red *n* = 21) vs. *cry-null* (red) flies (*n* = 12 UV; *n* = 17 blue; *n* = 12 red) in response to UV (365 nm LED, 150 μW/cm^2^) (*A*–*C*), blue (450 nm LED, 150 μW/cm^2^) (*D*–*F*), or red (630 nm LED, 150 μW/cm^2^) (*G*–*I*) light. (*A*, *D*, and *G*) Average changes in membrane potential with respect to light stimuli” for WT CRY control (black) and *cry-null* (red). (*B*, *E*, and *H*) FF change (during lights-on)/FF (dark baseline) for WT dCRY (black) and *cry-null* (red). (*C*, *F*, and *I*) FF change over time, during, and after light stimuli/FF (dark baseline) for WT dCRY (black) and *cry-null* (red). Data are represented as mean ± SEM; *P < 0.05; **P < 0.01 vs. WT dCRY.

The UV electrophysiological light responses recorded from WT dCRY fly l-LNv neurons show rapid onset and long-lasting membrane potential depolarization (Fig. 2*A*). The UV-light–evoked rapid membrane potential depolarization attenuates slowly and takes almost a full minute to return to baseline after light cessation (Fig. 2*A*). The UV light evokes increased FF during UV light stimulus (Fig. 2*B*), as reported previously (12). The post UV light stimulus increase in FF is surprisingly sustained when analyzed in 10-s bins for 10 to 20 s after UV light is turned off and returns to baseline FF within 30 to 60 s (Fig. 2*C*). Similarly, WT dCRY flies electrophysiologically respond to blue light with rapid increases in depolarization followed by a slow return to baseline membrane potential and increases in FF (Fig. 2 *D* and *E*), as reported (10). In WT dCRY flies, blue light also evokes sustained FF increases that return to baseline FF within a minute (Fig. 2*F*). Surprisingly, red light causes a measurable membrane-evoked potential depolarization (Fig. 2*G*). The red-light–evoked response is not as long lasting in WT dCRY flies as compared to the UV or blue-light–evoked responses (Fig. 2 *G* and *I*). Red light evokes minimal FF changes during the red light pulse, but the poststimulus probability of firing increases during the 10 s following the red light stimulus (Fig. 2 *H* and *I*). These results are unexpected because dCRY is reported as red light insensitive based on in vitro absorbance measurements using purified dCRY proteins. Alternatively, the highly sensitive light-evoked potential assay may be revealing opsin-based eye photoreceptor responses based on external photoreceptor light-dependent synaptic inputs to the LNv (31), although sustained light responses lasting up to 10 s duration post light stimulus are not an expected feature of image-forming opsin phototransduction.

We then measured light-evoked potential responses from l-LNv of *cry-null* negative control flies. Light-evoked potentials recorded from *cry-null* flies do not show depolarized membrane voltage changes in response to UV, blue, or red light (Fig. 2 *A*, *D*, and *G*). Surprisingly, light-evoked potentials recorded in *cry-null* l-LNvs in response to UV and blue light pulses show rapid hyperpolarization of the membrane potential followed by gradual recovery toward modestly depolarized values (Fig. 2 *A* and *D*). UV/blue-light–evoked membrane hyperpolarization in *cry-null* l-LNvs likely contributes to the absence of acute light-evoked FF increases during (but not after) UV and blue light stimuli in contrast to the robust light-evoked increases in FF in dCRY-expressing flies (Fig. 2 *B* and *E*). Tracking the delayed gradual repolarization following acute hyperpolarization, *cry-null* l-LNvs increase in FF for a 10-s poststimulus window after UV and blue lights are turned off (Fig. 2 *C* and *F*) and persist slightly above prelight baseline FF values for nearly a minute. The long poststimulus increases in FF evoked by UV, blue, and red light are much lower in amplitude in *cry-null* l-LNv than in WT dCRY-expressing neurons, but are still discernible (Fig. 2 *C*, *F*, and *I*). In contrast to WT, *cry-null* l-LNvs show no membrane potential depolarization during or after red light exposure (Fig. 2*G*). This supports the surprising possibility that light-activated dCRY in *Drosophila* neurons may express a FADH^•^ neutral semiquinone state flavin, which absorbs red light and signals an electrophysiological response. While red light does not reliably evoke changes in membrane potential in *cry-null* l-LNv, post red light FF increases during the 10-s bin and after red light exposure do not significantly differ from WT dCRY-expressing l-LNvs (Fig. 2 *H* and *I*).

Diphenyleneiodonium (DPI) is a fast-acting flavin-specific redox inhibitor that rapidly blocks dCRY-mediated electrophysiological response to blue light (11). In the absence of light, DPI has no effect on l-LNv firing frequency after 20 min of DPI incubation in the dark and thus appears to be light-activated in neurons (11). To confirm that blue-light–evoked responses in membrane potential change in WT dCRY-expressing l-LNv are mediated by dCRY, we measured the light-evoked potential response before and after DPI. The blue-light–evoked potential response is severely diminished in DPI-treated WT dCRY-expressing l-LNvs after light activation of the DPI flavin inhibitor, supporting the genetic findings that the electrophysiological light response seen in WT dCRY-expressing flies is mediated by the transgenic WT dCRY (*SI Appendix*, Fig. S3). DPI-induced dCRY inhibition of the blue-light–evoked potential does not show the initial hyperpolarization response seen in *cry-null* flies (*SI Appendix*, Fig. S3). We applied modified DPI/light-evoked potential and FF assays to examine DPI-inhibited electrophysiological responses for blue and red light. For the modified DPI-evoked potential assay all steps had a 90-s inter-light-pulse interval. We collected “pre-DPI” baseline light-evoked potentials averaged from 5 light pulses, added DPI, collected DPI light-evoked potentials averaged from 3 light pulses, and then collected post-DPI light-evoked potentials averaged from 5 light pulses. This allowed us to discern how rapidly DPI inhibitory effects occur, to resolve a more stable final inhibited state after 5 min of DPI, and we expected a monotonic inhibition of the light-evoked potential response from “pre-DPI” to “DPI” to “post DPI.” UV light responses were not measured because we could not hold stable recordings for the duration of the protocol. Using this protocol, we found monotonic DPI inhibition of the blue-light–evoked potential and that DPI significantly inhibits the initial light-evoked depolarization (*SI Appendix*, Fig. S4*A*) and FF during blue light stimulus (*SI Appendix*, Fig. S4*B*). Post blue light FF increases seen for the first 10 s after blue light do not significantly differ between the pre-DPI, DPI, and post-DPI conditions, but the longer sustained blue-light–evoked increases in FF are significantly lower compared to pre-DPI and post DPI (*SI Appendix*, Fig. S4*C*). Surprisingly, we also found monotonic DPI inhibition of the red light evoked potential and that DPI significantly inhibits the initial red-light–evoked depolarization (*SI Appendix*, Fig. S4*D*) and FF during red light (*SI Appendix*, Fig. S4*E*). Again, post red light FF increases seen for the first 10 s after red light do not significantly differ between the pre-DPI, DPI, and post-DPI conditions (*SI Appendix*, Fig. S4*F*). These surprising results further support the possibility that light-activated dCRY in *Drosophila* neurons express a FADH^•^ neutral semiquinone state. Due to the long duration of the protocol and prolonged DPI exposure for these assays, we tested the health of the preparation following the light-evoked potential series with a progressive 5-step ramp of 5-pA current injections followed by return to no current injection to measure the spontaneous action potential FF in the absence of light activation. A representative record shows that the preparation remains healthy as seen by robust increases in current-evoked FF and healthy spontaneous FF before and after the current injection ramp (*SI Appendix*, Fig. S4 *G* and *H*). In summary, WT dCRY mediates long duration complex electrophysiological UV, blue, and red light responses that significantly differ from neurons recorded in *cry-null* flies and acute DPI-treated brains.

### Tryptophan Residue Closest to dCRY FAD-Binding Site Is Critical for the Electrophysiological Response to UV, Blue, and Red Light.

**Fig. 3.**
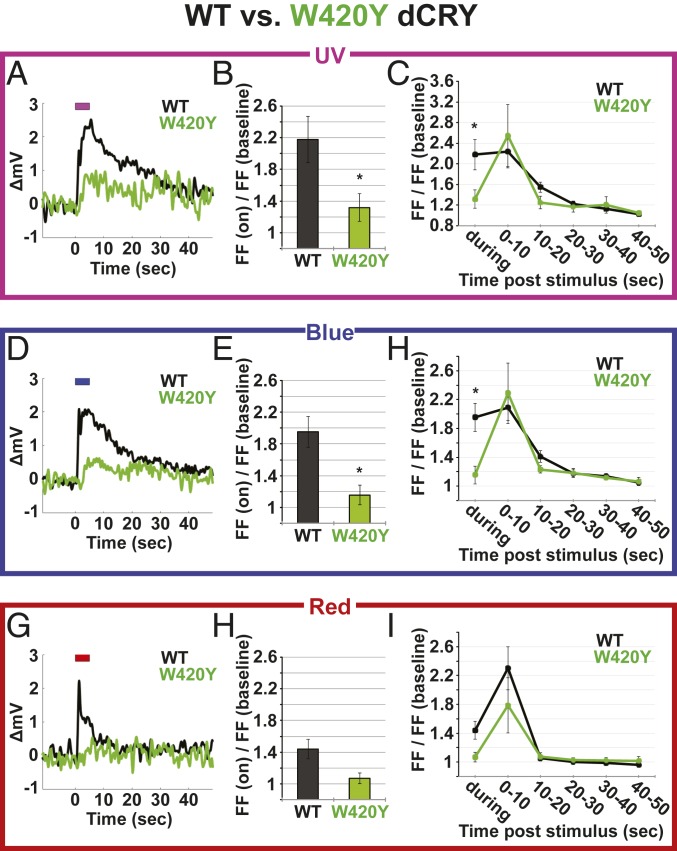
W420 tryptophan residue is critical for dCRY-mediated electrophysiological responses to UV, blue, and red light. l-LNv electrophysiological light responses of no mutation (WT dCRY) control (black) (WT dCRY: UV *n* = 19; blue *n* = 29; red *n* = 21) vs. *W420Y* mutant dCRY-expressing (red) flies (*n* = 5 UV; *n* = 8 blue; *n* = 6 red) in response to UV (365 nm LED, 150 μW/cm^2^) (*A*–*C*), blue (450 nm LED, 150 μW/cm^2^) (*D*–*F*), or red (630 nm LED, 150 μW/cm^2^) (*G*–*I*) light. (*A*, *D*, and *G*) Average changes in membrane potential in respect to light stimuli for WT dCRY control (black) and *W420Y* dCRY mutant (red). (*B*, *E*, and *H*) FF change (during lights-on)/FF (dark baseline) for WT dCRY (black) and *W420Y* dCRY mutant (red). (*C*, *F*, and *I*) FF change over time, during, and after light stimuli/FF (dark baseline) for WT dCRY (black) and *W420Y* dCRY mutant (red). Data are represented as mean ± SEM; *P < 0.05 vs. WT dCRY.

The high-resolution crystal structure of dCRY reveals a chain of Trp residues that may function as an electron transfer chain to mediate dCRY phototransduction along with a fourth Trp residue (20, 21). A functional Trp-triad molecular wire for redox transfer underlying the function of clock resetting has been reported for plant CRYs (19) and dCRY (24). Of these Trp residues, W420 is located the closest to dCRY FAD (Fig. 1). The dCRY W420Y mutation impairs both the rates and the extent of photoreduction in response to lower light intensities (24). Electrophysiological responses to low-intensity UV (365 nm LED, 150 μW/cm^2^), blue (450 nm LED, 150 μW/cm^2^), and red light (630 nm LED, 150 μW/cm^2^) were measured in l-LNv recordings from transgenic flies expressing the W420Y mutant dCRY. Compared to WT dCRY, l-LNv membrane potential depolarization during and following UV, blue, and red light exposures is severely attenuated in W420Y dCRY fly neurons (Fig. 3 *A*, *D*, and *G*). Furthermore, UV- and blue-light–evoked FF increases are significantly attenuated in W420Y dCRY neurons compared to WT dCRY (Fig. 3 *B* and *E*). Similar to *cry-null* flies, long duration FF changes following UV or blue or red light exposure do not significantly differ compared to FF change of WT dCRY-expressing flies (Fig. 3 *C*, *F*, and *I*), indicating again that light-evoked sustained increases in FF are not exclusively mediated by dCRY and likely interact with other light inputs. W420Y dCRY-expressing l-LNvs minimally respond to red light exposure and appear very similar to *cry-null* (Fig. 3 *G*–*I*). We conclude that the W420 residue closest to the dCRY-FAD binding site is important for dCRY-mediated light-evoked depolarization responses to UV, blue, and red light at relatively low light intensity.

### Tryptophan Residue Mutations More Distant from the dCRY FAD-Binding Site Have Minor Effects on Light-Evoked Electrophysiological Responses.

The rank order of proximity of residues in the Trp-triad to FAD in dCRY is W420, W397, and W342. In vitro, the W420Y mutation results in greater loss of photoreduction activity than W397Y or W342Y in purified mutant dCRYs exposed to moderate light levels (24). We recorded light-evoked electrical potentials and FFs from l-LNv of transgenic flies expressing W397Y or W342Y mutant dCRYs. W397Y dCRY-expressing l-LNv show modest attenuation of UV-light–evoked membrane depolarization (*SI Appendix*, Fig. S5*A*). FF of W397Y dCRY-expressing l-LNv robustly increases during UV light stimulus and does not significantly differ from WT dCRY-expressing l-LNvs (*SI Appendix*, Fig. S5*B*). Long-lasting UV-light–evoked FF changes in the W397Y dCRY mutant up to a minute following UV light exposure appear attenuated relative to WT dCRY control recordings but do not differ significantly (*SI Appendix*, Fig. S5*C*). The electrophysiological responses to low-intensity blue and red light recorded from W397Y dCRY mutant-expressing neurons are indistinguishable from those of WT dCRY control recordings (*SI Appendix*, Fig. S5 *D*–*I*). Similarly, the UV, blue, and red light electrophysiological responses recorded from W342Y dCRY mutant-expressing neurons are indistinguishable from those of WT dCRY control recordings (*SI Appendix*, Fig. S6), except for one long-term data point 20 to 30 s post stimulus (*SI Appendix*, Fig. S6*I*). Overall, W397 and W342 intermediate residues appear to be less critical for electrophysiological low-intensity light responses mediated by dCRY, in contrast to W420.

### The Distal W342 Tryptophan Residue Is Important for Circadian Photoentrainment of Locomotor Activity Rhythm.

**Fig. 4.**
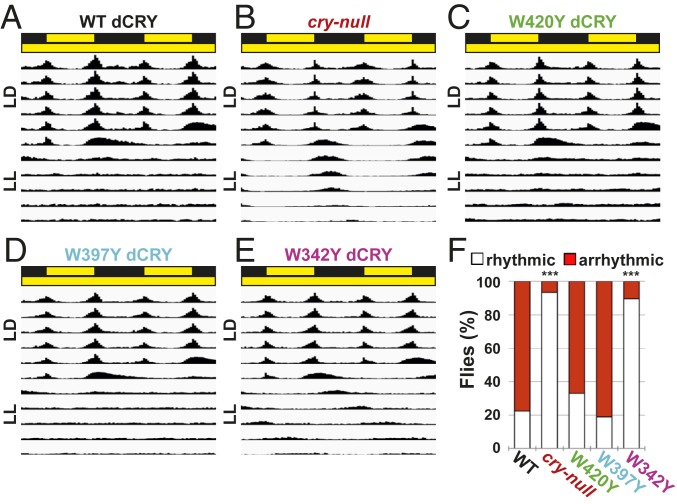
dCRY tryptophan 342 is critical for behavioral responses to constant moderate-low-intensity light. (*A*–*F*) Representative double-plotted actogram in standard 12 h:12 h moderate-low-intensity LD followed by LL (1,000 lx (1.5 W/m^2^), white light) for WT dCRY positive control (*n* = 147) (*A*), *cry-null* (*n* = 46) (*B*), and *W420Y* dCRY (*n* = 91) (*C*), *W397Y* dCRY (*n* = 90) (*D*), *W342Y* dCRY (*n* = 86) (*E*) transgenic mutant flies. (*F*) Percentages of rhythmic and arrhythmic flies in LL. Data are represented as mean ± SEM; ***P < 0.001 vs. WT dCRY.

CRY is a key modulator for circadian clock photoentrainment. Constant light exposure (LL) environmentally disrupts the circadian clock in many species, including *Drosophila*, and LL evokes behavioral arrhythmicity in a light-intensity–dependent manner (32, 33). Mutant *cry-null* flies remain behaviorally rhythmic in LL (30, 34) and thus are the basis of a clock-resetting behavioral assay to test dCRY Trp mutants in vivo. We tested whether WT dCRY, *cry-null* mutants, and transgenic dCRY mutant flies show impaired behavioral responses to LL after 12 h:12 h light:dark (LD) entrainment using moderately low (1,000 lx or ∼1.5 W/m^2^) and very low light (6 lx or ∼0.009 W/m^2^). Transgenic WT dCRY flies (positive control) and *cry-null* flies (negative control) and all dCRY Trp residue mutant flies entrain to LD (Fig. 4 and *SI Appendix*, Fig. S7). Transgenic flies expressing WT dCRY exhibit a predominately arrhythmic locomotion phenotype in moderate-low-intensity LL (1,000 lx), indicating that they express functional WT dCRY (Fig. 4 *A* and *F*). In contrast, *cry-null* flies maintain rhythmicity under LL (Fig. 4 *B* and *F*). Transgenic fly mutants that express either W420Y dCRY or W397Y dCRY are predominately arrhythmic in moderate-low-light-intensity LL (Fig. 4 *C*, *D*, and *F*). Thus, W420Y and W397Y dCRY mutant flies strongly resemble WT dCRY transgenic flies. In contrast, transgenic flies that express W342Y dCRY show nearly identical levels of high rhythmicity in moderate-low-intensity LL similar to *cry-null* flies (Fig. 4 *E* and *F*). Curiously, the W342Y dCRY mutants exhibit robust long periods in LL (period length: τ = 28.6 versus 24.9 for W342Y and *cry-null* flies, respectively), even though these flies share a common genetic background with the other flies tested (Fig. 4*E*). We repeated this behavioral assay under very low light intensity (1 to 10 lx). W342Y dCRY mutants and *cry-null* flies are both highly rhythmic in very-low-light-intensity LL, similar to their phenotype in moderate- low-light-intensity LL (period length: τ = 26.2 and 25.3 for W342Y and *cry-null* flies, respectively). W420Y dCRY mutants exhibit higher rhythmicity under very-low-light-intensity LL, compared to moderate-low-light-intensity LL (Fig. 4 and *SI Appendix*, Fig. S7). These differences suggest that for long-duration exposure to higher light levels in vivo, the W420Y mutants exhibit intensity-dependent compensation for dCRY photoreduction as suggested by previous in vitro and cellular studies (24).

## Discussion

Our previous work suggests that dCRY-mediated light-evoked changes in membrane electrical activity are mediated via a redox-based mechanism and intermolecular signal transfer to the HK redox-sensor potassium channel subunit (9–12). The present work supports and extends these findings. Compared to dCRY-mediated light-evoked electrical responses that are fast and relatively reversible, clock-resetting light responses appear to operate more slowly in terms of biological response time and are irreversible. There is strong consensus that light-evoked conformational change of dCRY contributes to light-induced degradation of dCRY and TIM (6, 14–16, 18, 24, 35). However, the biological significance of dCRY photoreduction in circadian clock resetting is less clear (17, 18, 24, 26) and was largely unknown for whole-animal in vivo studies until the present work.

Using electrophysiological assays, we found sustained light-evoked increases in membrane depolarization and long-lasting increases in FF following UV, blue, and red light stimuli that persist for up to a minute in WT dCRY-expressing l-LNv, but not in recordings from *cry-null* flies. Furthermore, UV- and blue-light–evoked potential recordings of *cry-null* l-LNv show a striking initial transient hyperpolarization in membrane potential. These results are consistent with an absence of short-wavelength–evoked changes in FF reported previously in *cry-null* flies (10, 12). The gradual resolution of hyperpolarization may account for the delayed increase in FF that is sustained for 10 s after cessation of UV and blue wavelength light exposures. This analysis of sustained light-evoked changes in membrane electrical potential and FF support previous findings that complex integration occurs between cell-autonomous and synaptic light inputs to the LNv (11, 12, 31). These findings point to detailed future studies to dissect the mechanistic contributions of opsins expressed in external photoreceptors and more recently discovered cell-autonomous photoreceptors such as Rhodopsin7 (36). One other intriguing possibility is that short wavelength light could cause the accumulation of reactive oxygen species generated by the dCRY photocycle, which could prolong the electrophysiological light response well beyond the duration of the light signal (37).

Surprisingly, red light evokes a significant dCRY-dependent depolarization that is absent both in genetic *cry-null* flies, following acute DPI treatment of WT-dCRY flies, and in W420Y dCRY mutant flies. These 3 lines of evidence suggest the possibility that neuronal dCRY could express a biologically active light-evoked FADH^•^ neutral semiquinone state. This merits further investigation to determine whether these red light responses are due to dCRY signaling or opsin signaling or the integration of both. As *cry-null* l-LNvs exhibit small and delayed increases in FF following red light exposure, the l-LNv likely has functional long wavelength light inputs other than dCRY. The most likely red-light-sensitive, non-CRY–mediated phototransduction input to the l-LNvs are red-sensitive opsins from the eyes and other external photoreceptors (31). Distinguishing dCRY vs. non-dCRY signaling requires careful control over light stimulus parameters. As dCRY light activation requires longer duration and higher intensity light stimuli than opsins, extremely short or low-intensity light stimuli fail to activate dCRY phototransduction (31). These differences could be exploited to further dissect opsin and dCRY electrophysiological photoresponses.

We generated transgenic flies expressing mutant dCRYs with residue tryptophan-to-tyrosine mutations in the classic Trp-triad chain. To measure light-evoked potential changes in neuronal membrane voltage in flies that express W420Y, W397Y, and W342Y dCRY mutants, we applied our highly sensitive light electrophysiological assays. Transgenic W420Y dCRY mutant flies show strongly reduced depolarization and significantly lower increases in FF in response to UV, blue, and red light relative to WT dCRY. However, the electrophysiological changes recorded from W420Y dCRY neurons do not entirely mimic the changes in light-evoked potential seen in *cry-null* mutant flies. Both W420Y dCRY flies and DPI flavin inhibitor-treated l-LNv neurons that express WT dCRY do not show the acute short wavelength light-evoked membrane hyperpolarization seen in *cry-null* mutant fly l-LNv recordings. Trp substitutions more distant to the dCRY FAD show no discernible defects in light-evoked membrane potential changes at the measured light stimulus intensity. These results do not rule out the possibility that W397 and W342 contribute to light-evoked electron transfer processes. Tyrosine is still redox sensitive but differs in protonation properties of its radical cation compared to Trp. Thus, these residues can still support electron transfer. Previous work shows that Trp residues closest to FAD and the protein surface are more sensitive to substitution than Trp middle residues as middle Trp residues can be “skipped” for electron transfer (24).

In contrast to the distinct effects of W420Y on light-evoked electrophysiological depolarization and rapid increases in FF, W342Y dCRY mutant flies show behavioral entrainment defects under LL, but have normal rapid electrophysiological light responses. These results suggest that W342 residue dCRY is important for circadian entrainment. The extent of light intensity and duration could account for these apparent differences by way of sensitivity of reduced flavin (17, 24, 26, 28). W420Y dCRY shows impaired light responses in electrophysiological assays, consistent with the short duration of light pulses used, which are expected to exacerbate decreases in photoactivation of W420Y. Motivated by the wide spectra of electrophysiological light responses from UV to red, we tested behavior responses to white light at similar intensities (1.5 W/m^2^). This light intensity was chosen in order to reduce dCRY signaling dimensionality from light intensity with duration to just duration. Further evidence for the importance of light intensity versus duration is shown in the LL behavior assay, for which mutation of the W342 residue shows greater impairment relative to the other Trp residues in the molecular chain. Higher yet still moderate-low-intensity/long-duration light is sufficient to mimic WT dCRY levels of arrhythmicity in W420Y and W397Y mutants, but LL-induced arrhythmicity is much less pronounced in these mutants under very-low-intensity/long-duration light. The results are consistent with the idea that long-duration light is required to release the CTT to trigger the proteolytic degradation process (24, 26). It is worth noting that under low- and very-low-intensity LL, W342Y mutants still exhibit unusually long LL periods not seen in *cry-null* flies. These results, along with the finding that there is no measurable correlation between expression levels and arrhythmicity, suggest that the W342Y defect in LL is not due to low protein levels (*SI Appendix*, Fig. S8). Furthermore, the W342Y mutant shows very robust responses for the electrophysiological assays. Low protein expression levels of the W342Y mutant may be indicative of destabilized conformation and changes in protein dynamics, which affect circadian function.

The distinct features of light-evoked slow clock resetting and fast neuronal electrophysiological depolarization both rely on photoinduced electron transfer along the Trp chain, but apparent mechanistic differences measured behaviorally in vivo and neuronal electrophysiological responses are likely due to how these processes are differentially sensitive to light intensity and duration. Furthermore, dCRY may be expressed in different cellular compartments. The dCRY-mediated neuronal light response is robust throughout the daytime when dCRY expression is low (10, 11) while the dCRY responsible for the clock-resetting mechanism is highly labile to light. The light responses of dCRY appear to integrate irradiance over longer periods, reminiscent of melanopsin signaling, to regulate behavioral arousal, environmental light choice, and photoentrainment (10–12, 38–40).

## Materials and Methods

Extended information on materials and methods are described in *SI Appendix*, including protocols for genetics, electrophysiology, optics, behavioral testing, and statistical analysis. Datasets described in this paper have been deposited in Harvard Dataverse, http://dataverse.harvard.edu (accession no. QLMWHR). Fly lines will be deposited in the Bloomington *Drosophila* Stock Center (https://bdsc.indiana.edu), which is searchable on FlyBase (http://flybase.org/). Fly lines can be obtained directly from the corresponding author until availability on Bloomington *Drosophila* Stock Center.

## Supplementary Material

Supplementary File
