# Supplementary material for: Distinct mechanisms of Drosophila CRYPTOCHROME-mediated light-evoked membrane depolarization and in vivo clock resetting
Source: Proc Natl Acad Sci U S A. 2019 Oct 28;116(46):23339–44. doi: 10.1073/pnas.1905023116 (PMC6859314; doi:10.1073/pnas.1905023116)
Supplement: Supplementary File [file pnas.1905023116.sapp.pdf]

Supplementary Information for

Distinct mechanisms of Drosophila CRYPTOCHROME-mediated light-evoked membrane depolarization and in vivo clock resetting

Lisa S. Baik, David D. Au, Ceazar Nave, Alexander J. Foden, Wendy K. Enrriquez-Villalva, and Todd C. Holmes[§]

[§]Corresponding author: Todd C. Holmes
Email: tholmes@uci.edu

This PDF file includes:

Materials & Methods
Figures S1 to S8
SI References

www.pnas.org/cgi/doi/10.1073/pnas.1905023116

Materials & Methods

Fly Lines

- ***cry-null:*** *w; pdfGAL4-p12c;cry^{01}*
- ***WT dCRY and dCRY Mutants:*** *Cry24-gal4*; *UAS-eGFP-(WT or Mutant dCRY); cry^{01}* (see below for details).
- ***No UAS negative control***: *cry24GAL4;;cry^{01}*

Transgenic Fly Line Construction

We generated a vector from synthetic DNA (Genescript) containing a full *Drosophila* cryptochrome sequence, in frame with *eGFP* (Addgene). Point mutations of CRY were introduced into wild type *dCRY* tagged with *eGFP* in the *UAS-pJFRC7* vector (1). Wild-type and mutant *dCRY-eGFP* constructs in the pJFRC7 vector allow for controlled insertion into same genomic location (a specific PhiC31 genomic site. when injected into fly embryos (1), thus allowing for direct experimental comparison of resultant transgenic fly lines. The pJFRC7 vectors containing wild-type control and experimental mutant *dCRY-eGFPs* were injected into fly embryos, which were screened for eye color as evidence of successful transgenesis. Resultant transgenic flies were isogenized by backcrossing with *w^{1118}* flies for a minimum of 6 generations.

Point mutations were introduced to the template vector using QuikChange™ II Site-Directed Mutagenesis Kit (Agilent). Following primers were designed as recommended in the QuickChange™ protocol:

- ***W420Y*, with codon change of TGG to TAC**:
  agctggatacccacatgtagttgccagcgcaga and gtctgcgctggcaactacatgtgggtatccagct
- ***W397Y*, with codon change of TGG to TAC**:
  gctgcagtccatgctcgtagctctgccacaaacc and ggtttgtggcagagctacgagcatggactgcagc
- ***W342Y*, with codon change of TGG to TAC**:
  cgttcggcttagcgtacgggatgctcaggcag and ctgcctgagcatcccgtacgctaagccgaacg

As quality control, mutant vectors were sequenced before proceeding (Retrogen, Inc.). WT (no mutation) and mutant *dCRY* vectors were injected into *Drosophila* embryo (BestGene) to generate the U*AS-eGFP-dCRY(mutation)* transgenic flies. *UAS-eGFP-dCRY* (*WT* or *mutant dCRY*) flies were crossed into *cry^{01}* background and again with *cry24-gal4* to generate the final transgenic mutant line expressing *WT* or *mutant dCRY* under cry-driver in a *cry-null* background:

- **WT dCRY (no mutation) control:** *cry24-GAL4*; *UAS-eGFP-CRY; cry^{01}*
- **W420Y dCRY:** *cry24-gal4*; *UAS-eGFP-CRY(W420Y); cry^{01}*
- **W397Y dCRY:** *cry24-gal4*; *UAS-eGFP-CRY(W397Y); cry^{01}*
- **W342Y dCRY:** *cry24-gal4*; *UAS-eGFP-CRY(W342Y); cry^{01}*

Fluorescence Imaging & Quantification

All flies were kept under 12 hr: 12 hr light: dark (LD) schedule until 3 days before dissection, at which point the flies were kept in constant darkness (DD) for 3 days. On the third day of DD at circadian time (CT) 21– 24, adult male fly brains were dissected in 1x PBS solution under dim red light and immediately mounted in VECTASHIELD (Vector Labs). To minimize introducing variance by circadian timing or experimental differences, all flies were entrained and dissected at the same time and days. Dissections were carried out at the same time for all genotypes tested and were repeated over 3 total experimental repeats. Brains were imaged with Zeiss LSM 700 confocal microscope. Fluorescence was quantified with ImageJ by selecting all regions of CRY-expressing/GFP-positive neurons and obtaining the mean intensity, and normalized to mean intensity level obtained by selecting background region outside of the brain. Fluorescence of cry-expressing neurons were not quantified for the no-UAS negative control flies (cry24GAL4;;cry^{01}) as there were no clear GFP expression present to be able to identify those neurons.

Electrophysiology

Whole-cell current-clamp recordings were carried out from protocols adapted from (2-4). Adult male (3-5days post-eclosion) fly brains were dissected in external recording solution. l-LNv were subjected to whole-cell current-clamp with external solution: 122mM NaCl, 3mM KCl, 1.8mM CaCl2, 0.8mM MgCl2, 5mM glucose, 10mM HEPES, 7.2 pH, and 250-255mOsm; internal solution: 102mM Kgluconate, 17mM NaCl, 0.085mM CaCl2, 1.7mM MgCl2 (hexahydrate), 8.5mM HEPES, 0.94mM EGTA, 7.2pH, and 232-235mOsm.

Custom-ordered multichannel LED source (Prizmatix/Stanford Photonics, Palo Alto, CA) fitted to the Olympus BX51 WI miscrscope was used for all optics using electrophysiology recordings. LED peak wavelengths are as follows: UV (365 nm) blue (450 nm), and red (630 nm), and all exposures were set to intensity of 150μW/cm^2. Light intensities were determined by a Newport 842-PE Power/Energy meter. Each LED was triggered on and off for each sweep with TTL pulses programmed by pClamp (Molecular Dynamics) data acquisition software. Each color pulse was 5 seconds long. Each light pulse was preceded by minimum 50 second pre-pulse dark baseline, and there was 95 second inter-pulse intervals between each light exposure from there on, with 5-10 times of each color exposed per cell. All sweeps containing each light exposure recordings were averaged and baseline was adjusted to pre-pulse signal. Furthermore, Gaussian and Butterworth filters were applied to the averaged signals using the ClampFit 10 software (Molecular Dynamics).

For DPI recordings, diphenyleneiodonium chloride (Sigma) was diluted in the external recording solution at 16.8μM concentration. An initial 5 light exposure sweeps

of pre-DPI recording were obtained, followed by a 3 sweeps obtained immediately after the addition of DPI (<5 minutes), and 5 sweeps of post-DPI injection (>10 minute). Finally, the recording was complete with a 100 second current-step protocol of +5pA/10 second up to +20pA, followed by a return to no current injection.

Behavior

Adult male flies were selected at 2-4 days post-eclosion then loaded in individual locomotor activity tubes. Locomotor activity of individual flies was measured using the TriKinetics Locomotor Activity Monitoring System via infrared beam-crossing recording total crosses in 15 or 30 min bins. Flies were initially entrained in 12 hr: 12 hr light:dark (LD) condition for 7 days, then they were exposed to 7 days of constant light (LL) condition at varying light intensities (e.g., 1-10 lux, 1000 lux) measured by a LI-COR LI-250A light meter with a LI-210R photometric sensor. Light intensities were adjusted using GamColor CineFilter 1516 .6 neutral density filters wrapped around the light bulbs to achieve lower light intensity lights. Addition of light bulbs allowed to achieve higher light intensities. Actograms were generated using Clocklab software. Average activity eduction graphs, % rhythmic flies, and its statistics were measured using FaasX software, then graphed using Microsoft Excel.

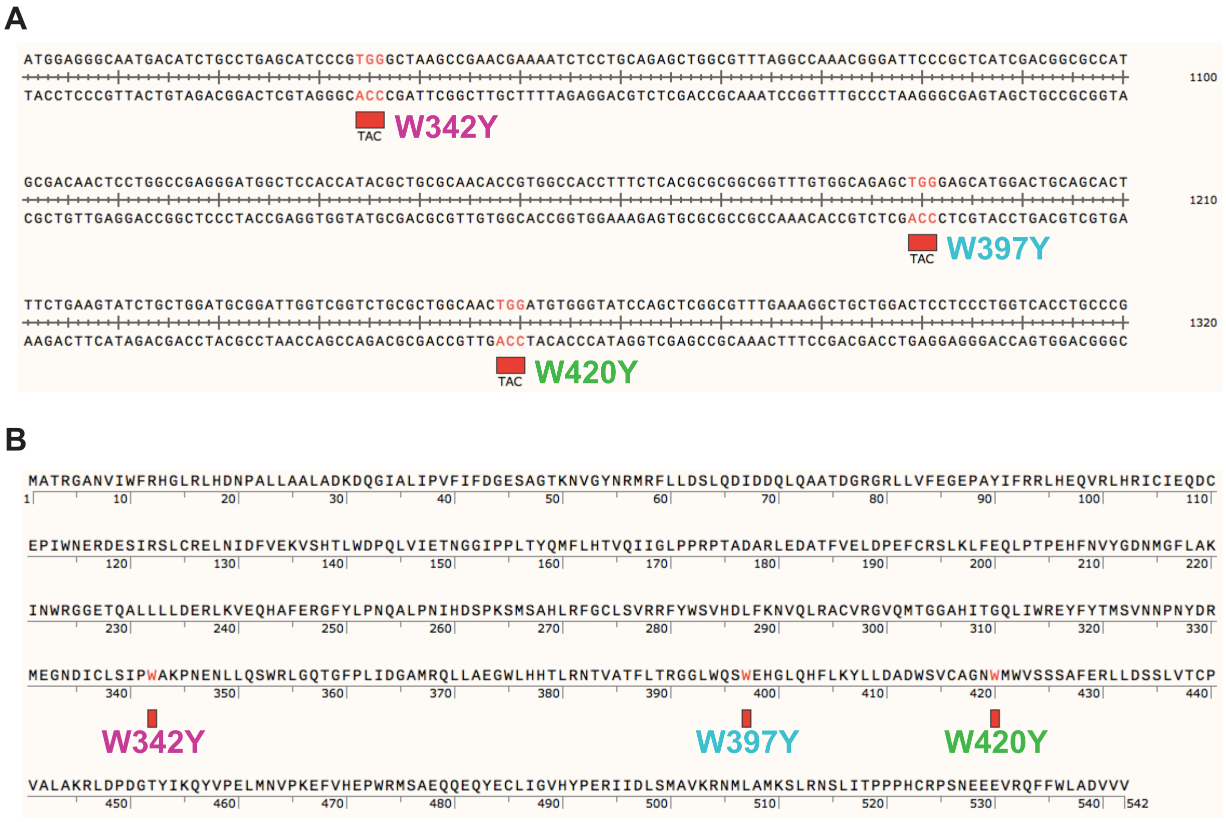

Figure S1. Map of site-directed mutagenesis in *cryptochrome*.
(A) Codon sequence of *Drosophila cryptochrome* showing sites of sequence change in tryptophan to tyrosine residues. **(B)** Protein sequence of *Drosophila CRYPTOCHROME* indicating (red) sites of mutagenesis.

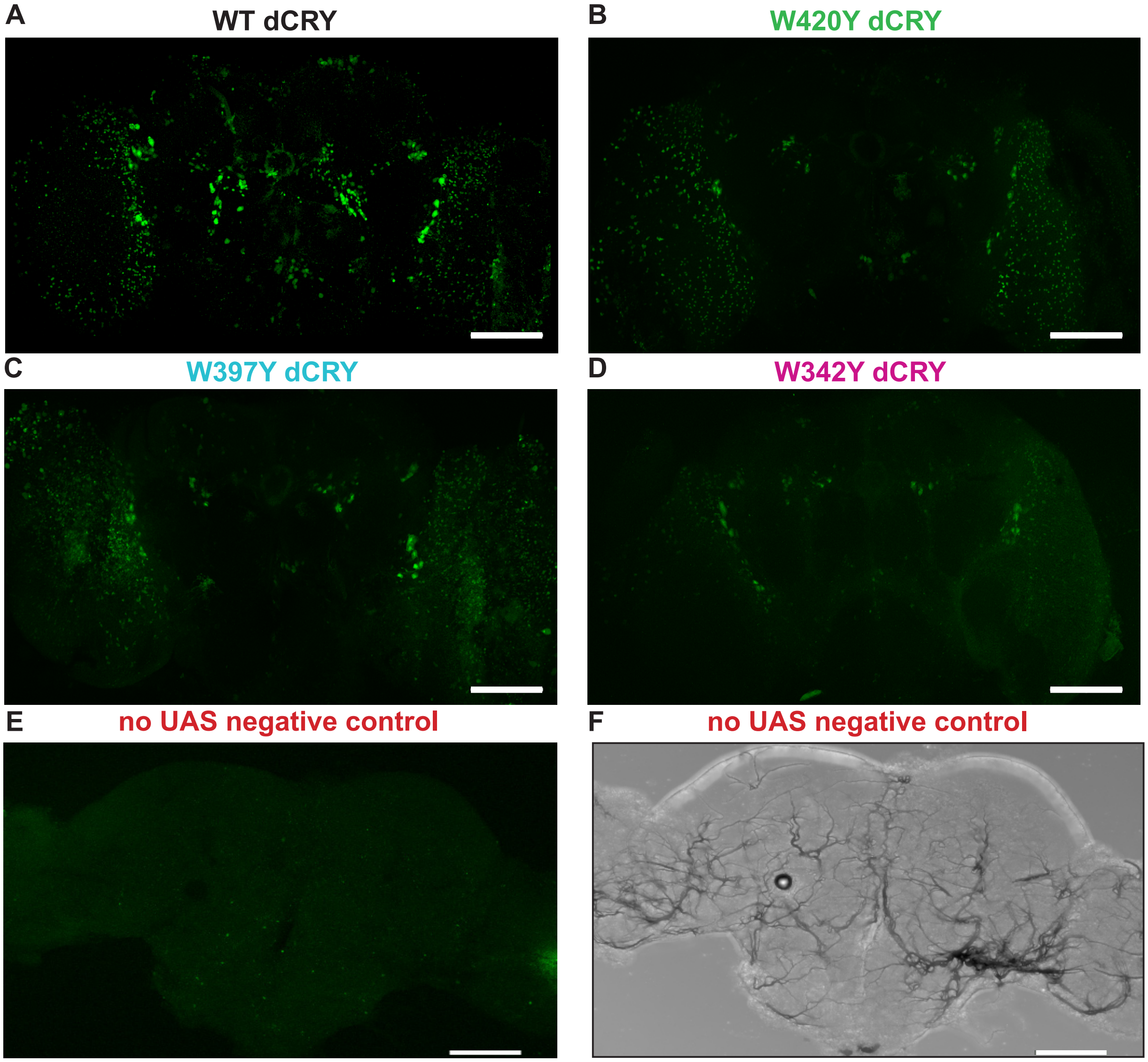

Figure S2: Confocal imaging confirming expression of eGFP-tagged transgenic *Drosophila* CRYPTOCHROME (dCRY) in a adult transgenic fly brains.
Confocal images of freshly dissected brains showing eGFP-tagged expression of transgenic WT (n=11) **(A)**, W420Y (n=10) **(B)**, W397Y (n=11) **(C)**, and W342Y (n=11) **(D)** dCRYs, as well as *cry24GAL4;;cry*^{01} no UAS negative control (n=10) **(E)**. **(F)** bright field image of the same *cry24GAL4;;cry*^{01} no UAS negative control brain shown in **(E)**. Scale bar indicates 100µm.

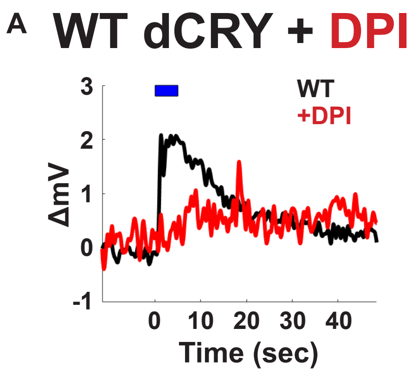

Figure S3. FAD-specific redox inhibitor DPI abolishes WT dCRY electrophysiological response to short wavelength light.
(A) l-LNv electrophysiological light responses of changes in membrane potential for WT dCRY control (black trace) in standard external solution vs. administration of DPI (red trace) blue light stimuli (450 nm LED, 150μW/cm^2, n=5).

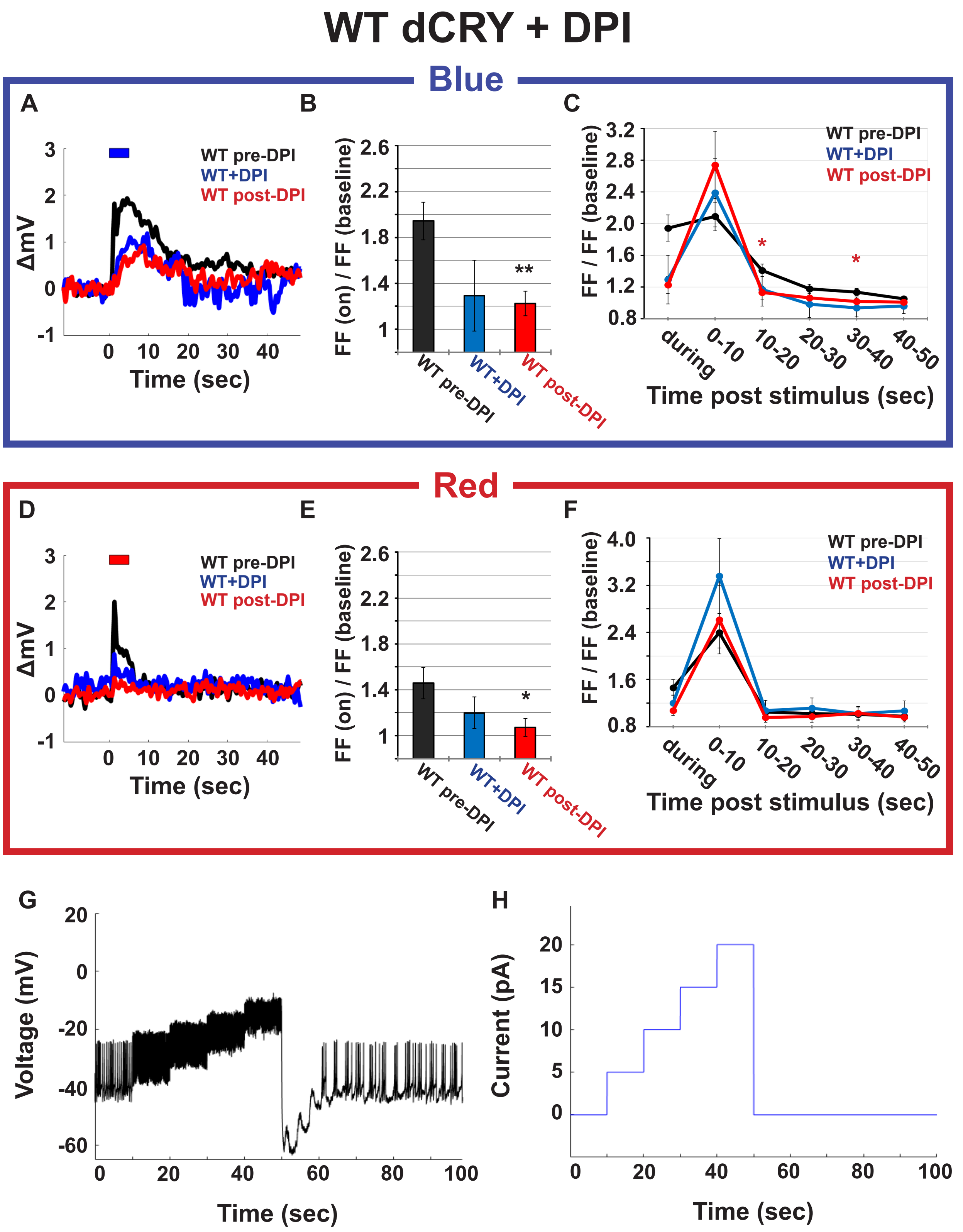

Figure S4. FAD-specific redox inhibitor DPI abolishes WT dCRY electrophysiological response to short wavelength light.

l-LNv electrophysiological light responses of changes in membrane potential for WT dCRY control in standard external solution before DPI administration (black) vs. immediately following DPI administration (blue), and post-DPI administration (red) with **(A-C)** blue light stimuli (450 nm LED, 150μW/cm^2, n=7) or **(D-F)** red light stimuli (640 nm LED, 150μW/cm^2, n=5). **(A, D)** shows average changes in membrane potential in respect to pre-DPI administration (black), immediately following DPI (blue), and post-DPI administration (red). **(B, E)** Firing frequency (FF) change (during light on)/ FF (dark baseline) for pre-DPI administration (black), immediately following DPI (blue), and post-DPI administration (red). **(C, F)** FF change over time, during and after light stimuli/ FF (dark baseline) for pre-DPI administration (black), immediately following DPI (blue), and post-DPI administration (red). **(G)** Representative current step recording trace of **(H)** current injections steps +5pA/10 second up to +20pA, followed by a return to no current injection. Data are represented as mean ± S.E.M. *$p < 0.05$ vs. pre-DPI administration.

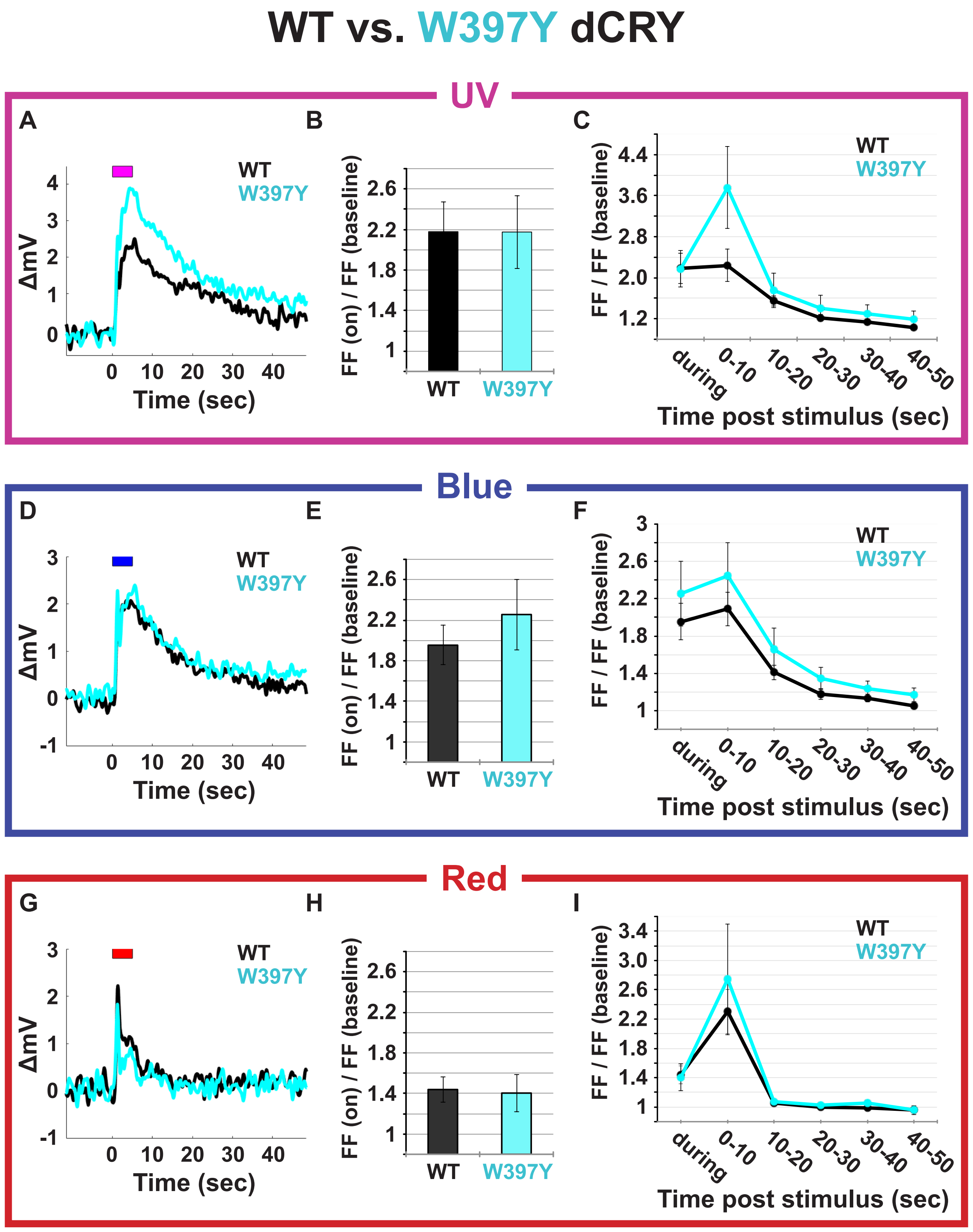

Figure S5. W397Y Tryptophan residue mutation does not affect dCRY-mediated electrophysiological response to UV and blue light

l-LNv electrophysiological light responses of no mutation (WT dCRY) control (black) (WT dCRY: UV n=19; blue n=29; red n=21) vs. *W397Y* mutant dCRY-expressing (red) flies (n=7 UV; n=11 blue; n=7 red), in response to UV (365 nm LED, 150μW/cm^2) **(A-C)**, blue (450 nm LED, 150μW/cm^2) **(D-F)**, or red (630 nm LED, 150μW/cm^2) **(G-I). (A, D, G)** shows average changes in membrane potential in respect to light stimuli for WT dCRY control (black) and *W397Y* dCRY mutant (red). **(B, E, H)** Firing frequency (FF) change (during light on)/ FF (dark baseline) for WT dCRY (black) and *W397Y* dCRY mutant (red). **(C, F, I)** FF change over time, during and after light stimuli/ FF (dark baseline) for WT dCRY (black) and *W397Y* dCRY mutant (red). Data are represented as mean ± S.E.M.

WT vs. W342Y dCRY

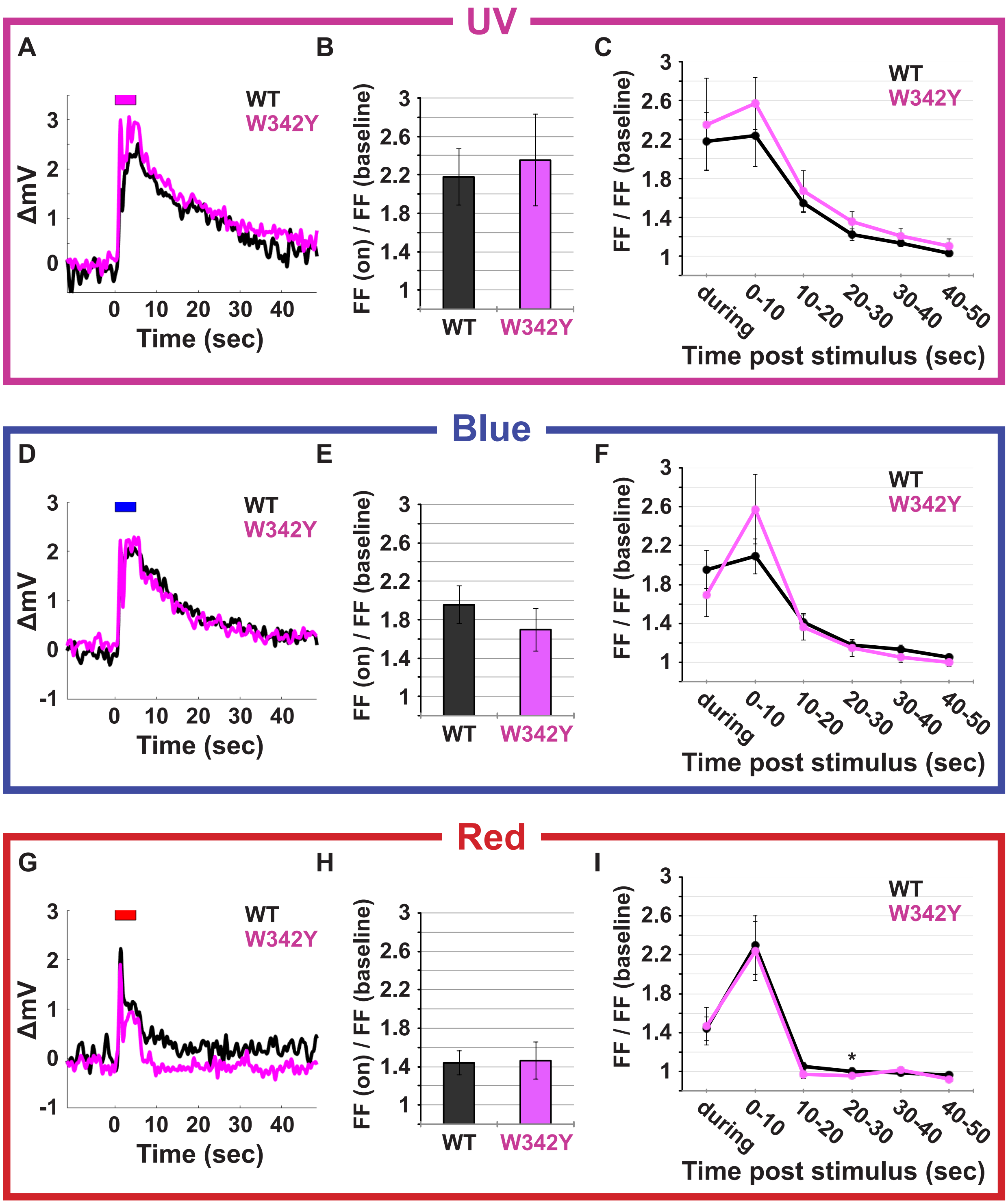

Figure S6. W342 Tryptophan residue is not required for dCRY-mediated electrophysiological response to UV and blue light

l-LNv electrophysiological light responses of no mutation (WT dCRY) control (black) (WT dCRY: UV n=19; blue n=29; red n=21) vs. *W342Y* mutant dCRY-expressing (red) flies (n=12 UV; n=16 blue; n=12 red), in response to UV (365 nm LED, 150μW/cm^2) **(A-C)**, blue (450 nm LED, 150μW/cm^2) **(D-F)**, or red (630 nm LED, 150μW/cm^2) **(G-I). (A, D, G)** shows average changes in membrane potential in respect to light stimuli for WT dCRY control (black) and *W342Y* dCRY mutant (red). **(B, E, H)** Firing frequency (FF) change (during light on)/ FF (dark baseline) for WT dCRY (black) and *W342Y* dCRY mutant (red). **(C, F, I)** FF change over time, during and after light stimuli/ FF (dark baseline) for WT dCRY (black) and *W342Y* dCRY mutant (red). Data are represented as mean ± S.E.M. *p < 0.05 vs. WT dCRY.

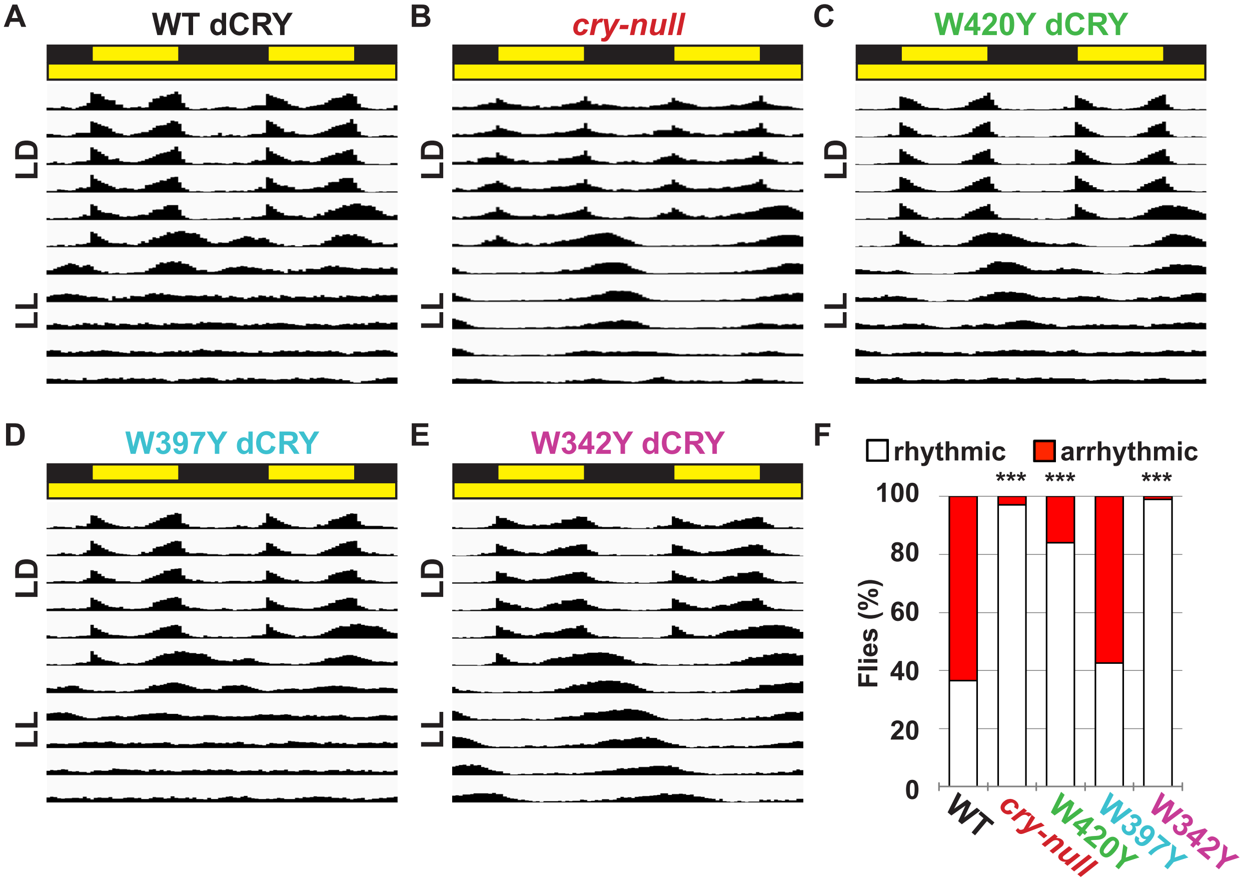

Figure S7. Tryptophan mutant dCRY flies have defective circadian photoentrainment under very dim light.
(A-F) Representative double-plotted actogram in standard 12hr: 12hr very low intensity light: dark (LD) followed by constant light (LL) (1-10 lux, white light) for WT dCRY positive control (n=148) **(A)**, *cry-null* (n=68) **(B)**, and *W420Y* dCRY (n=88) **(C)**, *W397Y* dCRY (n=94) **(D)**, *W342Y* dCRY (n=92) **(E)** transgenic mutant flies. **(F)** Percentages of rhythmic and arrhythmic flies in LL. Data are represented as mean ± S.E.M. *p<0.05, ***p < 0.001 vs. WT dCRY.

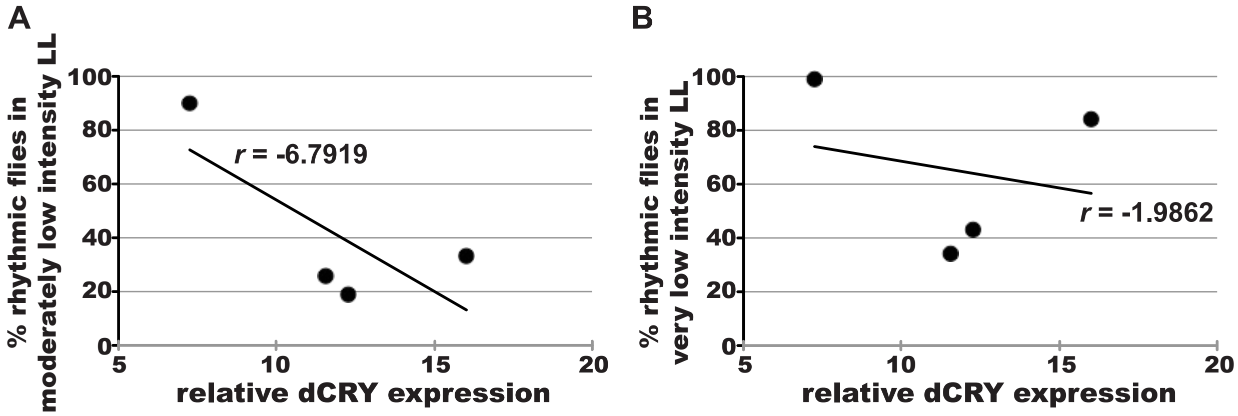

Fig. S8. Transgenic dCRY expression and % rhythmicity of LL behavior do not show clear correlation.
(A, B) Lack of correlation between eGFP expression levels versus % rhythmicity of behavior under moderately low intensity light LL (correlation coefficient= -6.7919) **(A)** and very low intensity light LL **(B)**. Also refer to Figures 1, 4 and S8

Supplementary Information References

1. Pfeiffer BD*, et al.* (2010) Refinement of tools for targeted gene expression in Drosophila. *Genetics* 186(2):735-755.
2. Baik LS*, et al.* (2017) CRYPTOCHROME mediates behavioral executive choice in response to UV light. *Proceedings of the National Academy of Sciences of the United States of America* 114(4):776-781.
3. Fogle KJ*, et al.* (2015) CRYPTOCHROME-mediated phototransduction by modulation of the potassium ion channel beta-subunit redox sensor. *Proceedings of the National Academy of Sciences of the United States of America* 112(7):2245-2250.
4. Fogle KJ, Parson KG, Dahm NA, & Holmes TC (2011) CRYPTOCHROME is a blue-light sensor that regulates neuronal firing rate. *Science (New York, N.Y.)* 331(6023):1409-1413.
